# William H. Pitt

**Published:** 1909-09

**Authors:** 


### OBITUARY

---

William H. Pitt, who died at his home in Friendship, N. Y., Sunday morning, July 11, 1909, lived a life of rare usefulness, attaining a commanding position among the scientific men of the country. As a geologist, he was an authority in America, and he lived to see millions made from his chemical discoveries.

Professor Pitt came to Buffalo in the early '70s to take charge of the physic classes at the high school. He had rare success as a teacher, and no member of the faculty was so well liked by the pupils as Professor Pitt, the kind, gentle, unostentatious gentleman who was so absorbed in his chemical researches. After eighteen years of patient classroom work at the high school, he was able to retire to his home in Friendship, with a competency.

He crowded much activity in to his scientific life. Born at Short Tract, Allegany County, in 1831, he prepared himself for school during the long winter when there was no work to do on the farm. He worked his way through Alfred University, being graduated in 1857. He then went to Union College, taking a diploma in 1860. From 1861 to 1865, he was principal of the Spencer High School and of the Angelica Academy. In 1867, he became superintendent of education at Warren. He became principal of the Friendship Academy in 1869, staying there until he came to Buffalo. In 1881-2, he was the state analyst of food and drugs and in 1884, he was appointed to the chair of chemistry and physics at Niagara University, giving many lectures there every year during its existence.

In 1879, he was granted the degree of M.D. by the University of Buffalo and in 1886, Alfred University conferred the degree of Ph. D. upon him. He was a member of the Buffalo Society of Natural Sciences since 1872, contributing to the society's publications.

